# Nutritional state variations in a tropical seabird throughout its breeding season

**DOI:** 10.1007/s00360-022-01456-3

**Published:** 2022-09-13

**Authors:** Miriam Lerma, Nina Dehnhard, José Alfredo Castillo-Guerrero, Guillermo Fernández

**Affiliations:** 1 Posgrado de Ciencias del Mar y Limnología, Universidad Nacional Autónoma de México, Ciudad Universitaria, Coyoacán, 04510 Ciudad de Mexico, México; 2 Research and Technology Center (FTZ), University of Kiel, Hafentörn 1, 25761 Büsum, Germany; 3 Norwegian Institute for Nature Research (NINA), Høgskoleringen 9, NO-7034 Trondheim, Norway; 4 Departamento de Estudios para el Desarrollo Sustentable de la Zona Costera, Centro Universitario de la Costa Sur, Universidad de Guadalajara, San Patricio-Melaque, Municipio de Cihuatlán, Jalisco, 48980 México; 5 Unidad Académica Mazatlán, Instituto de Ciencias del Mar y Limnología, Universidad Nacional Autónoma de México, Mazatlán, Sinaloa 82040 México

**Keywords:** Seabirds, Sex, Reproduction, Triglycerides, Cholesterol, ß-hydroxybutyrate

## Abstract

Individual body condition is frequently used to explain differences in foraging and breeding ecology in seabirds. However, little is known about the covariations of body mass with the nutritional state of animals as measured through plasma metabolites and how these different measures vary between and within individuals during breeding. Here, we assessed intra-individual variations of plasma metabolites (triglycerides, cholesterol, protein, and ß-hydroxybutyrate concentrations) and in body mass of Blue-footed boobies (*Sula nebouxii*) throughout their breeding season 2011–2012 in Isla El Rancho, Mexico. We found breeding-stage and sex-specific variations in individuals’ plasma metabolite concentrations, but these did not mirror variations in body mass. Before egg-laying, females had higher triglycerides, cholesterol, and protein concentrations than males. In contrast, males used their nutritional reserves (higher ß-hydroxybutyrate concentrations) more than females during the breeding season (except for early chick-rearing). At the individual level, males gained weight during the breeding season, whereas females lost weight. We also found that between-individual differences in plasma metabolite concentrations and changes in body mass were not consistent throughout the breeding season, while individual body mass was significantly repeatable. This study contributes to a better understanding of seabird breeding ecology and physiology by showing that sex-specific breeding roles might highly influence the nutritional state. Similar patterns might occur in other seabird species, helping to explain why we can find stage- and sex-specific foraging behaviors even in monomorphic species.

**Supplementary Information:**

The online version contains supplementary material available at 10.1007/s00360-022-01456-3.

## Introduction

Body condition may affect the foraging decisions of animals (Saraux et al. 2011), and the nutritional state of individuals may be decisive for animals to reproduce or not, and how much to invest in any given reproductive attempt (Wendeln and Becker 1996; Ellis et al. 2000; Dehnhard et al. 2015; González-Medina et al. 2020). In long-lived species such as seabirds, which can breed for many years, adults should carefully trade-off their survival with reproductive investment (sensu ‘the prudent parent hypothesis’; Drent and Daan 1980). However, individuals differ in their morphological and physiological characteristics, such as body size, body condition, or fat reserves. Some of this heterogeneity may reflect differences in individual quality based on genetic variation (Jenouvrier et al. 2018; Vedder and Bouwhuis 2018) and/or behavioral and environmental interactions such as an individual’s ability to forage (Lescroël et al. 2010). Individual specialization in specific foraging tactics or diets could also contribute to variation in foraging success between individuals (Tarroux et al. 2020) although such links may not hold over long periods (Dehnhard et al. 2016; Phillips et al. 2017).

Although seabirds have been extensively used as model species to investigate breeding investment strategies based on body condition and individual quality (e.g. Weimerskirch 1998; Jenouvrier et al. 2018; González-Medina et al. 2020), information on the individual variation of the nutritional state of seabirds throughout breeding is limited. In one example, adult Scopoli’s shearwaters *Calonectris diomedea* showed distinct patterns of body mass and plasma metabolites as indicators of nutritional state throughout the breeding season from egg formation to chick-rearing (Navarro et al. 2007). Most studies that evaluated the energetic cost of breeding used changes in body condition or body mass alone. However, birds may compensate for variations in their body mass by increasing their foraging effort and energy intake (Ewing et al. 2005) or reducing their energetic demands by decreasing their breeding investment (González-Medina et al. 2015; Kidawa et al. 2015; Storey et al. 2017). Thus, considering plasma metabolites as indicators of nutritional state, in addition to changes in body mass, can provide valuable information. High plasma triglycerides, cholesterol, and total protein are associated with building energetic reserves and individuals in good condition. In contrast, ß-hydroxybutyrate is frequently associated with fasting periods, and high concentrations of this metabolite are typical for individuals with poor body condition (see Table 1 for more detailed reference information).

**Table 1:** Plasma metabolites and the hypotheses linked to the nutritional state of Blue-footed boobies **(*Sula nebouxii*)** during the breeding season

Plasma metabolite	Biological interpretation	References	Hypothesis			
			Pre-laying	Incubation	Early rearing	Late rearing
Triglycerides	Food absorption and good alimentation. Recent energy intake	Jenni-Eiermann and Jenni (1994); Jenni and Schwilch (2001)	$\uparrow$	$\downarrow$	$\downarrow$	$\uparrow$
Cholesterol	Increase in food ingestion. Feeding success	Alonso-Alvarez and Ferrer (2001); Alonso-Alvarez et al. (2002)	$\uparrow$	$\downarrow$	$\downarrow$	$\uparrow$
Total proteins	Ingestion of proteins. Increases in body fat levels	Jenni-Eiermann and Jenni (1998); Smith and McWilliams (2009)	$\uparrow$	$\downarrow$	$\downarrow$	$\uparrow$
ß-hydroxybutyrate	Catabolization of body stores. Fasting periods or a starvation state	Jenni-Eiermann and Jenni (1998); Guglielmo et al. (2005)	$\downarrow$	$\uparrow$	$\uparrow$	$\downarrow$

Pre-laying: the period when parents were defending a territory and on courtship. Incubation: the period immediately after laying the first egg until the hatching of the first chick. Early rearing: the period corresponding to rearing 4–5 weeks old chicks. Late-rearing: the period of rearing 10–12 weeks old chicks. Predictions: $\uparrow$: higher concentrations of this metabolite, $\downarrow$ : lower concentrations

The Blue-footed booby (*Sula nebouxii*) is an ideal seabird species to study nutritional state variations (using plasma metabolites) and body condition metrics (using body mass and changes in their body mass). This species can be easily captured; one can collect enough blood to analyze plasma metabolites, and many aspects of their breeding biology are well known. This species is socially monogamous, and both parents take turns incubating the eggs and feeding the chicks until they fledge (Nelson 1978). In addition, Blue-footed boobies are sexually dimorphic (Nelson 1978), with females being heavier (30–32%) and larger (5–10%) than males. Females in this species may store nutritional reserves that can be used when necessary (Velando and Alonso-Alvarez 2003) and may increase their contribution to chicks (Guerra and Drummond 1995). In contrast, the males’ contribution to provisioning is lower and remains relatively stable after the second week of age of its chicks (Guerra and Drummond 1995). Therefore, it has been suggested that males operate at a physiological maximum (Velando and Alonso-Alvarez 2003) but can adjust their parental care and foraging according to the demands of chicks (González-Medina et al. 2015). Previous research indicated that body condition and plasma triglyceride concentrations affect mate choice in Blue-footed boobies, i.e., pairs mated assortatively based on these traits (González-Medina et al. 2020). Further, elevated plasma triglyceride concentrations in females were linked to a high-quality diet and increased breeding performance (González-Medina et al. 2018).

Our current study aimed to investigate the intra-seasonal variation in the nutritional state (using several plasma metabolites) and body mass at the individual level throughout the breeding season. Based on studies with other seabird species, we expected that individuals exhibit (i) higher blood levels of triglycerides, proteins, and cholesterol, and a higher body mass at the pre-laying stage (Wendeln and Becker 1996); (ii) higher blood levels of ß-hydroxybutyrate (indicating the use of their nutritional reserves) during incubation and during chick-rearing, and a reduction in their body mass due to the fasting periods and accumulative effects of breeding (Dearborn 1996; Navarro et al. 2007); and (iii) higher blood levels of triglycerides, proteins, and cholesterol and an increase in their body mass reflecting a recovery from breeding at the end of the breeding season, due to a reduction in provisioning the chick (Weimerskirch and Lys 2000). In addition, based on studies with other sulids (see Table 2), we expected sex-specific differences, with females having elevated levels of plasma metabolites (triglycerides, cholesterol, and total proteins) than males during breeding. We also expected body mass changes to be weaker than changes in plasma metabolites in both sexes because Blue-footed boobies might compensate for the energetic cost of breeding without exhibiting changes in their body mass. Finally, given that plasma metabolites are a snapshot compared to body mass, we assessed whether individuals remained consistent in their plasma metabolites levels and body mass.

**Table 2:** Studies on nutritional state and/or body condition on sulids

Species & site(s)	Breeding stage	Sex	References
Blue-footed booby			
El Rancho	F: C > I, R, L^1,2^	F > M^2^	This study
El Rancho	C	F > M^1^	González-Medina et al. (2020)
Lobos de Tierra	R	F > M^2^	Velando and Alonso-Alvarez (2003)
Isla Isabel	C = R^3^	F > M^2,4^	Wingfield et al. (1999)
Brown booby			
Christmas Island	I = R^5^	F > M^5^	Dehnhard and Hennicke (2013)
Nazca booby			
Punta Ceballos	R > L^2^	F > M^2^	Apanius et al. (2008)
Masked booby			
Rapa Nui	I < R^2^	F > M^2^	Lerma et al. (2020)
Red-footed booby			
Mozambique Channel	C, I, L > R^6^	F > M^6^	Lormée et al. (2003)
Australasian gannet			
Pope’s eye & Point Danger	I = R^2^	F > M^2,7^	Angel et al. (2015)
Phillip Bay	I = R^2^	U	Ewing et al. (2005)
Northern gannet			
Bass Rock, Grassholm, Great Saltee & Rouzic	R	F > M^8^	Grecian et al. (2019)
Cape gannet			
Malgas & Bird Island	R^9,10^	U	Moseley et al. (2012)
Malgas Island	R^11^	U	Grémillet et al. (2016)

Species included are the following: Blue-footed booby *Sula nebouxii*, Brown booby *Sula leucogaster*, Nazca booby *Sula granti*, Masked booby *Sula dactylatra*, Red-footed booby *Sula sula*, Australasian gannet *Morus serrator*, Northern gannet *Morus bassanus*, and Cape gannet *Morus capensis*. Comparisons of body condition are made between *F*: females, *M*: Males, *U*: Undetermined; and between breeding stages: *C*: courtship/pre-laying period, *I*: Incubation, *R*: Early rearing, *L*: Late rearing

Body condition definitions vary between studies. Therefore, the data presented correspond to different methods, including the following: ^1^nutritional state (using plasma metabolites); ^2^body mass; ^3^testosterone levels in plasma; ^4^corticosterone levels in plasma; ^5^body mass divided by the cube of wing chord length; ^6^residual body mass corrected by size using tarsus for females and wing length for males; ^7^body mass corrected using wing and ulna length; ^8^scalated mass index using bill length; ^9^body mass divided by wing length; ^10^breast muscle thickness; ^11^body mass divided by wing chord

## Methods

### Fieldwork

The study was conducted at Isla El Rancho (25°10’N, 108° 23’W), a sandy ~ 400 ha island at the northern mouth of Bahía Santa María, which is a coastal lagoon system in Sinaloa, Mexico. The island holds a colony of ~ 3,000 pairs of Blue-footed boobies (Castillo-Guerrero et al. 2014) and was visited every two weeks from 12 December 2011 to 12 May 2012. Nests were randomly selected and monitored throughout the breeding season. The following four phases of breeding were considered: pre-laying (when parents were defending a territory and showing courtship behavior), incubation (from laying the first egg until the hatching of the first chick), early rearing (when chicks were 4–5 weeks old), and late rearing of chicks (when chicks were 10–12 weeks old). There was considerable variability in laying dates for those individuals who laid an egg, with individuals laying their first egg between 7 December and 26 January. Although our goal was to monitor the same nest throughout the breeding season, the number of sampled individuals decreased as the breeding season progressed because birds were either not found at the nest, could not be captured, or had experienced breeding failure.

All individuals were captured at their nest with a hand net from a 1-m distance. Individuals were weighed and measured, and blood samples were collected. The individuals were weighted in a bag with an electronic balance (± 1 g). Ulna length was measured using a steel ruler (± 1.0 mm), bill length (from nasofrontal hinge to the top), and tarsus were measured using calipers (± 0.1 mm). The sex of the individuals was registered based on the size, the pupil, and the voice of the individuals: females are larger, have an irregular edge pupil, and a rougher call than males (Nelson 1978). A total of 21 females (ulna length 208 ± 4 mm, culmen length 110 ± 2 mm, and tarsus length 55 ± 1 mm) and 23 males (ulna length 191 ± 3 mm, culmen length 104 ± 2 mm, tarsus length 51 ± 1 mm) were weighed and measured. From these individuals, 20 birds were weighed again at each one of the following three breeding stages (*N* = 80 measurements in total). From all individuals, a blood volume ≤ 0.5 mL was extracted with a 25G syringe. A total of 42 individuals were blood-sampled during courtship, 28 during incubation, 24 during early rearing, and 25 during late rearing. The time of day when the sample was collected and the total handling time (< 15 min from the moment of capture to release) was recorded. Blood samples were centrifuged at 10,000 rpm for 10 min to separate plasma from red cells. Plasma and red cells were stored at – 20 °C until subsequent laboratory analyses.

In the laboratory, the following five metabolites were quantified: total triglycerides, free glycerol, cholesterol, total proteins, and ß-hydroxybutyrate. Real triglyceride metabolite concentrations were calculated by subtracting free glycerol from total triglycerides. Reagents used were from commercial kits adapted to small-sample volumes (Albano et al. 2011): total triglycerides (triglyceride plus free glycerol, 2.3 µL plasma, 230 μL reagent, Menagent, Menarini diagnostics); free glycerol (2.5 μL plasma, 200 μL reagent, Sigma); cholesterol (2.3 μL plasma, 230 μL reagent Menagent, Menarini diagnostics); total protein (2.5 μL plasma, 250 μL reagent, Menagent, Menarini diagnostics); and ß-hydroxybutyrate (5 μL plasma, 100 reagent A, 100 μL reagent B, Enzytec, Diagnostic Systems). Total triglycerides, cholesterol, and total proteins metabolite concentrations were determined using a multiparametric autoanalyzer (Falcor 360; Menarini Diagnostics, Barcelona, Spain), whereas free glycerol and ß-hydroxybutyrate metabolite levels were determined using a microplate spectrophotometer (BioTek, Winooski, VT, USA) via endpoint assay. All samples were duplicated, and the analyses were calibrated using commercial calibration kits and control reference serums accordingly. Inter- and intra assay coefficients of variations were below 10%. Not all blood samples had enough volume to analyze all the plasma metabolites. Thus, priority was given as follows: triglycerides (*n* = 116), cholesterol (*n* = 114), ß-hydroxybutyrate (*n* = 108), and total proteins (*n* = 104).

Studies on seabirds often use body mass or several other body metrics such as body mass change (Δ_BM_) to evaluate changes in body condition during the breeding season (see Table 2). Body mass and Δ_BM_ were used to assess which metrics (if any) mirrored the plasma metabolites’ results best. We defined body mass as the weight of the individuals in grams at the moment of the measurement (*n* = 118) and body mass changes (Δ_BM_) as the individuals’ weight difference from their mean at the moment of the measurement (*n* = 80 measurements of 20 individual birds). The mean was calculated using the body mass from all the times the individual was weighted.

### Statistical analyses

Statistical analyses were performed using R 4.0.3 (R Development Core Team 2020). According to their breeding stage, we employed generalized linear mixed effect models (GLMMs) using the package ‘lme4’ (Bates et al. 2015). Single models were performed separately for each plasma metabolite (triglycerides, cholesterol, proteins, and ß-hydroxybutyrate) and body condition metric (body mass and Δ_BM_). The models were constructed with the plasma metabolites or body condition metrics as dependent variables; sex and breeding stage and the interaction of sex and breeding stage were used as fixed factors. All models included bird ID as a random effect to account for pseudo-replication. We used single-term deletions and the Kenward–Roger approximation for degrees of freedom to test the significance of fixed effects of the model using the package ‘lmerTest’ (Kuznetsova et al. 2017). Initial models for plasma metabolite (triglycerides, cholesterol, proteins, and ß-hydroxybutyrate) analyses were constructed, including ulna and body mass to account for the possible effect of size, the handling time to account for any variation due to manipulating the animals, and time of the day as numeric to account for a possible effect of foraging activity periods. Ulna size, body mass, handling time, or time of the day were not significant (*p* > 0.05) and not included in the plasma metabolite analyses. Initial models for body condition metrics (body mass and Δ_BM_) analyses were constructed, including ulna size to account for the possible effect of size and time of the day as numeric to account for a possible effect of foraging activity periods. Models did not retain ulna size, likely because body mass variations were not related to the size of the individuals (Fig. S1). The models retained time of the day (*p* < 0.05) as individuals were heavier later in the day, likely due to foraging activity. Thus, the models for body condition metrics included the time of the day as a random effect. Model assumptions were tested as described in Zuur et al. (2009). Post-hoc tests to compare groups were performed using the function ‘difflsmeans’ from the package ‘lmerTest’ (Kuznetsova et al. 2017).

To investigate the between-individual consistency, also commonly referred to as ‘repeatability’, we employed longitudinal models using each plasma metabolite concentration (triglycerides, cholesterol, proteins, and ß-hydroxybutyrate) or body condition metric (body mass and Δ_BM_) as the dependent variable separately, and individual as a random factor in the package ‘rptR’ (Stoffel et al. 2017). This package calculates variances between and within individuals and produces a repeatability estimate (*R*) value (± SE), 95% confidence intervals (CI) obtained from 1000 bootstrap iterations and *p* values. The repeatability estimate values are between 0 and 1, with values closer to 1 representing higher repeatability and values closer to 0 representing lower repeatability. Repeatability is defined as the proportion of the total variance accounted for by differences among groups (Nakagawa and Schielzeth 2010). This means that repeatability is a function of both the within-group and between-group variance. Repeatability can be expected to be low if either the within-individual variation is high and/or the between-individual variation is low (Nakagawa and Schielzeth 2010). Only individuals sampled at all four breeding stages were included in the repeatability analysis (*n* = 20).

To test if the nutritional state of Blue-footed boobies was decisive in continuing reproduction or not, we assessed the relationship between the nutritional state (using plasma metabolite concentrations) and body mass and breeding continuity (0 = abandoned breeding, 1 = continued breeding), using generalized linear models (binomial distribution and logit link) separated by sex. In addition, we tested for differences during courtship, hatching, or chick-rearing for these analyses, depending on when the breeding failure occurred. Analyses were made separately by plasma metabolite concentration (triglycerides, cholesterol, proteins, and ß-hydroxybutyrate) or body mass as the continuous variable.

## Results

### Intra-seasonal variations

**Fig. 1:**
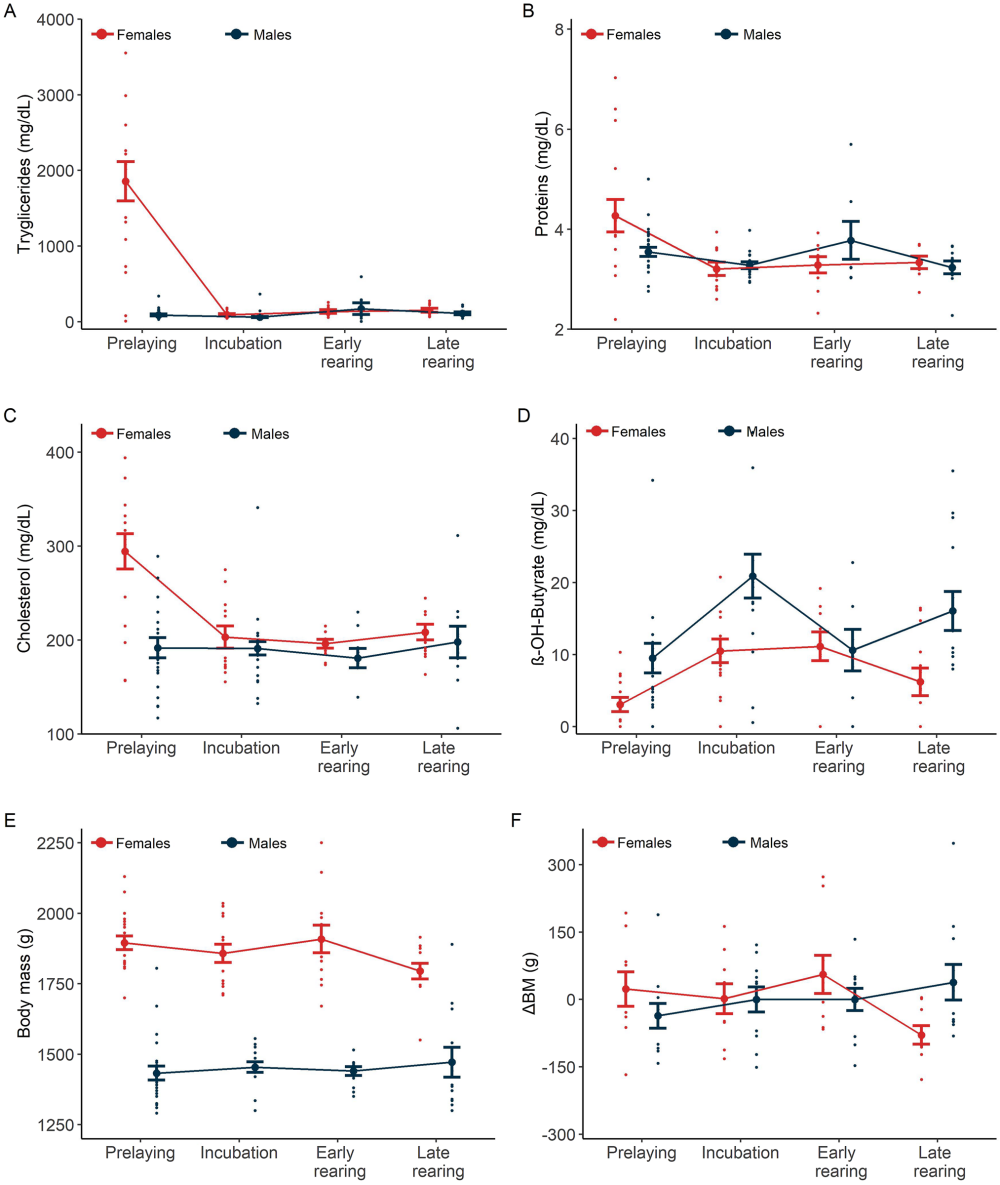
Plasma metabolite concentrations, body mass, and body mass changes (Δ_BM_) of female and male Blue-footed boobies (*Sula nebouxii*) during the breeding season 2012 at Isla El Rancho, Mexico. Plasma metabolites presented are triglycerides (*n* = 116), total proteins (*n* = 104), cholesterol (*n* = 114), and ß-hydroxybutyrate (*n* = 108). Body mass (*n* = 118) and Δ_BM_ (*n* = 80) are included. Means (± standard error) and individual values are presented

The interaction between sex and breeding state was significant for triglycerides (*F*_1,3_ = 111.06, *p* < 0.01), cholesterol (*F*_1,3_ = 8.69, *p* < 0.01), total proteins (*F*_1,3_ = 4.35, *p* < 0.01), and ß-hydroxybutyrate (*F*_1,3_ = 3.15, *p* = 0.03). Females had significantly higher triglycerides, cholesterol, and total proteins than males during the pre-laying period (Post-hoc test *p* < 0.05). However, the levels of these metabolites were similar between both sexes during incubation, early and late chick-rearing (*p* > 0.05, Fig. 1). For ß-hydroxybutyrate, the pattern was different, males had significantly higher concentrations of this metabolite during the breeding season (excepting early chick-rearing) than females (Post-hoc test *p* < 0.05, Fig. 1).

For body mass, the interaction of sex and breeding stage was not significant (*F*_1,3_ = 1.78, *p* = 0.07). After removal of the interaction term, breeding stage was not significant (*F*_1,3_ = 0.60, *p* = 0.62), but sex was significant (*F*_1,3_ = 224.60, *p* < 0.01). Females were heavier than males at all breeding stages (Fig. 1). For body mass changes (Δ_BM_), comparisons were only made for weighted individuals in the four breeding stages because this measurement is based on average weight. Hence, 80 measurements of 20 individuals (female = 9, male = 11) were included in this analysis. For Δ_BM_, the interaction of sex and breeding stage was significant (*F*_1,3_ = 288.59, *p* < 0.01). Females were lighter than their average at the end of the season, whereas males showed the opposite pattern by being heavier than their average at the end of the breeding season (Fig. 1).

### Individual consistency

**Fig. 2:**
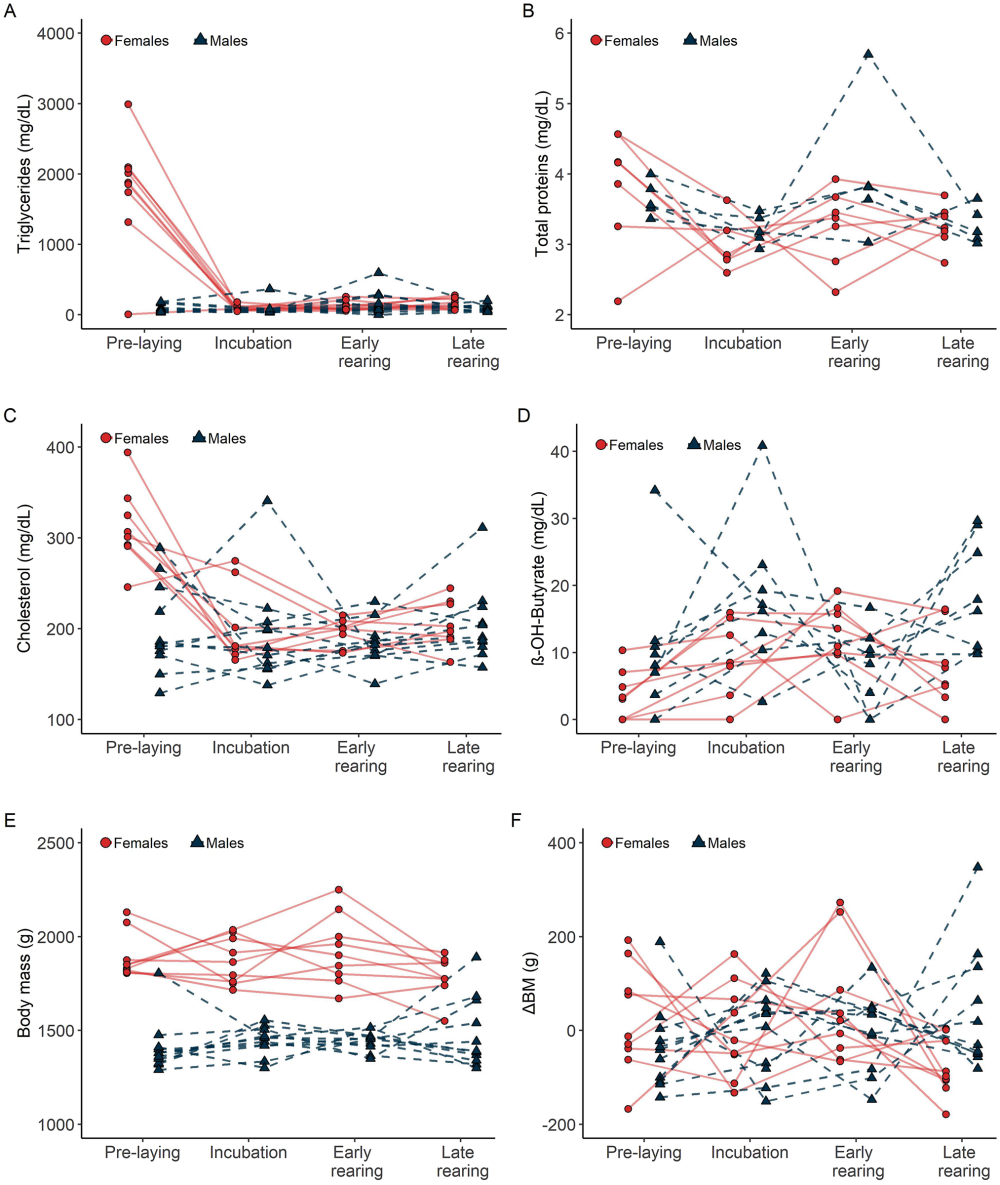
Individual plasma metabolite concentrations, body mass, and changes in body mass (Δ_BM_) of Blue-footed boobies (*Sula nebouxii*) during the breeding season 2012 at Isla El Rancho, Mexico. Only individuals that were sampled at all four stages of breeding are included. Plasma metabolites presented are triglycerides (female = 9, male = 11), total proteins (female = 7, male = 5), cholesterol (female = 8, male = 11), and ß-hydroxybutyrate (female = 8, male = 8). Body mass and Δ_BM_ are included (female = 9, male = 11). Individual concentrations and trajectories are presented (females in circles and filled lines, males in triangles and dashed lines)

We found a low repeatability value for triglycerides (*R* = 0 ± 0.05, CI = 0, 0.18, *p* = 0.50), total proteins (*R* = 0 ± 0.07, CI = 0, 0.21, *p* = 1.00), cholesterol (*R* = 0.10 ± 0.09, CI = 0, 0.31, *p* = 0.19), ß-hydroxybutyrate (*R* = 0 ± 0.06, CI = 0, 0.20, *p* = 0.50), and Δ_BM_ (*R* = 0 ± 0.06, CI = 0, 0.18, *p* = 1.00), indicating that there were no consistent between-individual differences in these metabolites and Δ_BM_ throughout the breeding season (Fig. 2). In contrast, we found a high repeatability value for body mass (*R* = 0.78 ± 0.06, CI = 0.64, 0.86, *p* < 0.05), indicating that there are consistent differences in weight between individuals throughout the breeding season.

### Breeding continuity

From the 42 individuals selected at the beginning of the breeding season, 15 females laid an egg, 19 males started to incubate eggs, and the rest abandoned breeding. Individuals that abandoned breeding did not differ in their nutritional state or body condition metrics during courtship from those that laid eggs (Table 3). From the 28 individuals monitored during the incubation period, eggs from only three individuals did not hatch (1 female, 2 males). Individuals with hatching eggs did not differ in their nutritional state or body mass from those whose eggs did not hatch (Table 3). Finally, from the 24 individuals monitored throughout the breeding season, while comparing individuals with chicks with those without chicks (1 female, 1 male), we found no difference in the nutritional state or body mass of both groups (Table 3).

**Table 3:** Results from generalized linear models testing for breeding continuity (binomial distribution: 0 = abandon breeding, 1 = continue breeding) based on plasma metabolites and body mass separated by sex and depending on when the breeding failure occurred (courtship, incubation, or chick-rearing)

	Courtship			Incubation			Chick-rearing		
	df	*X*^2^	*p*	df	*X*^2^	*p*	df	*X*^2^	*P*
Females									
Triglycerides	18	0.84	0.39	13	− 0.82	0.41	11	0.60	0.55
Total proteins	14	− 0.89	0.37	12	0.00	1.00	11	0.001	1.00
Cholesterol	15	0.003	0.99	13	− 0.92	0.35	11	− 1.15	0.25
ß-hydroxybutyrate	16	0.02	0.99	13	1.01	0.31	10	0.00	1.00
Body mass	18	− 0.60	0.55	13	− 0.42	0.68	11	− 0.57	0.57
Males									
Triglycerides	22	0.95	0.34	13	− 0.18	0.86	11	0.58	0.56
Total proteins	20	0.20	0.84	12	0.48	0.63	8	0.65	0.52
Cholesterol	22	1.40	0.16	13	1.05	0.29	11	− 0.19	0.84
ß-hydroxybutyrate	18	− 0.18	0.86	13	1.30	0.19	8	0.56	0.88
Body mass	22	0.68	0.50	13	− 0.68	0.50	11	− 0.59	0.55

## Discussion

In line with our predictions, plasma metabolite concentrations in breeding adults varied according to breeding stage and sex. Females were in a higher absorptive state than males, particularly during the pre-laying period (more elevated triglycerides, proteins, and cholesterol concentrations). The females’ absorptive state is probably related to the higher acquisition of nutritional reserves to satisfy gonadal growing and egg production during pre-laying (Moreno 1989). This absorptive state during pre-laying may reflect a widespread pattern in female birds. For example, Navarro et al. (2007) found similar patterns of elevated triglycerides, proteins, and cholesterol in female Cory’s shearwaters during pre-laying compared to egg-laying, and similar results have been found in other species of sulids (see Table 2). However, in Cory’s shearwaters, triglycerides, proteins, and cholesterol were elevated in both sexes again during hatching and chick-rearing. In contrast, our results showed that both sexes had similar concentrations of these metabolites during those stages. Similarly, in Brown skuas (*Stercorarius antarcticus*), triglycerides, total proteins (Graña Grilli et al. 2018), and cholesterol (Ibañez et al. 2021) remained invariant from incubation to late chick-rearing without sex differences. Thus, species-specific differences in the breeding biology, body reserves, and possibly diet are potential explanations for the physiological patterns among seabird species. In addition, it might be critical that female Blue-footed boobies are in a higher absorptive state to build reserves to endure the breeding season. In Blue-footed boobies, females supply more food during the chick-rearing period (Guerra and Drummond 1995), and this high effort in provisioning chicks also helps explain their body mass reduction at the late stages of breeding. This result contrasts with Cory’s shearwaters, where both sexes contribute equally to chick provisioning, and the body mass of both sexes increases until hatching, to then decline (Navarro et al. 2007).

In order to cover their energetic requirements, female Blue-footed boobies might forage further from their colonies (Weimerskirch et al. 2009), dive deeper or consume larger prey items (Zavalaga et al. 2007) than males. In Blue-footed boobies, males spend more time in the colony earlier in the season to ensure their territory (Osorio-Beristain and Drummond 2001). In contrast, females may forage further from the colony in areas with less competition and higher prey availability as in other sulids (Lerma et al. 2020; Roy et al. 2021). In a related species, the Australasian gannet (*Morus serrator*), each sex targeted prey that differs in nutrient content (Machovsky-Capuska et al. 2016). However, previous studies found no dietary segregation between the sexes in Blue-footed boobies (González-Medina et al. 2017, 2020). Nevertheless, it is possible that females have a higher assimilation rate during the pre-laying period and are freer to forage than males due to their differential breeding roles, thus foraging at specific areas and prey species to satisfy their nutritional needs. In addition to the physiological demands around egg production as discussed above, this pattern could explain why the differences occurred during the pre-laying period and not during the other breeding stages. Females’ needs to satisfy their energetic requirements, particularly during the pre-laying period, may also occur in monomorphic species, providing additional explanations of why we can find sex-specific foraging strategies (Lewis et al. 2002; Ismar et al. 2017) and differences in body condition in species such as gannets (Table 2).

Blue-footed booby males, the smaller sex, made more extensive use of their body reserves (higher ß-hydroxybutyrate) than females in all breeding stages except early chick-rearing. This result agreed with the suggestion that in boobies, males work at their physiological maximum (Velando and Alonso-Alvarez 2003; Dehnhard and Hennicke 2013). The more extensive use of body reserves in males may result from males performing shorter foraging trips to spend more time defending a territory and avoiding extra-pair paternity during the pre-laying period (Osorio-Beristain and Drummond 2001). In other seabird species, males return sooner to the breeding colony (Phillips et al. 2017) or remain close to their colonies than females before breeding (Pistorious et al. 2015), including in some booby species (Roy et al. 2021). Remaining closer to their colonies, an early return, and shorter foraging trips at the onset of breeding may prevent Blue-footed booby males from gathering a buffer of nutritional reserves and thus need to catabolize their body stores during the breeding season.

None of the body condition metrics mirrored the nutritional state measured from plasma metabolites. In Blue-footed boobies, changes in body mass (Δ_BM_) were the only body condition metric sensitive to significant variations throughout breeding. Unexpectedly, males increased their average body mass at the end of breeding, whereas females experienced a reduction in their average body mass. In Blue-footed boobies females may increase their feeding frequency to chicks sharply at least until the chicks are 20 days old to then decline, while the food mass provided increases progressively at least until the chicks are 35 days old (Guerra and Drummond 1995). The cost of feeding their growing chicks might thus reduce the females’ body mass during this period. In contrast, in Red-footed boobies, the males’ body condition declines significantly during the chick-rearing period (see Table 2), but males spend more time at sea during this period (Lormée et al. 2005). In Blue-footed boobies, males likely reduce their energetic expenses and increase their foraging effort, prioritizing self-provisioning to recover their body mass. Nonetheless, differences in food among Blue-footed and Red-footed booby colonies might significantly explain the differences in body mass recovery at the end of the season. Other seabird species recover their mass at the end of the breeding season (Weimerskirch and Lys 2000), possibly reducing carry-over effects on subsequent breeding seasons (Harrison et al. 2011). Interestingly, in Blue-footed boobies, males may take a sabbatical year after a demanding breeding event (Velando et al. 2010). Thus, the recovery at the end of the breeding season may be crucial for males. In a related species, the Masked booby, females reach and stay in more productive waters during the pre-breeding period than males (Roy et al. 2021). Consequently, females might regain their lost condition in these areas, whereas males might be compensating for poor foraging conditions as early as the end of current breeding.

Importantly, we found that only body mass but not the plasma metabolites were repeatable. In the case of body mass, heavier birds during the pre-laying period continue to be heavier in subsequent breeding stages. In contrast, the nutritional state at the beginning of the breeding season was not maintained at later breeding stages. Potential reasons could be the variability of the plasma metabolites. For example, outside the egg-laying phase—which goes along with the activation of nutritional reserves and thus metabolic processes, as discussed above—the birds’ physiology may be more sensitive to the uptake of nutrients from the birds’ diet and diet composition. At the study site, the Blue-footed boobies’ prey is diverse (González-Medina et al. 2017), and given that prey items have different nutritional components (Machovsky-Capuska et al. 2016), the diet composition may influence the physiological variation, including, e.g., triglyceride concentrations, as has been shown before in Blue-footed boobies (González-Medina et al. 2018). However, the reason for the variations in the nutritional state is unclear, and it is not new that it is difficult to capture the complete picture to understand differences in physiological metrics (Fowler et al. 2018), particularly in field studies (Mitchell et al. 2021).

Plasma metabolites are commonly used as indicators of individual quality, a term that is linked to long-term heterogeneity between individuals (Wilson and Nussey 2010). Previous studies in Blue-footed Boobies confirmed a relationship between female triglyceride concentrations (sampled during courtship) and the summed triglycerides of a pair to their reproductive performance (González-Medina et al. 2018; 2020). González-Medina et al. (2020) also found a correlation between the summed triglycerides of a pair measured during courtship and those measured during chick-rearing. However, the fact that plasma metabolites were not repeatable within individuals throughout the entire breeding season in this present study, and thus between-individual differences in these plasma metabolites do not remain consistent throughout the entire breeding season, highlights the need to exercise caution when using plasma metabolites as indicators of individual quality (sensu Fowler et al. 2018; Mitchell et al. 2021). Possibly, triglyceride levels during courtship reflect true individual quality, while this is not necessarily the case at later stages, e.g. due to different nutrient uptake among individuals (as discussed above).

Contrary to our expectations and previous findings in Blue-footed boobies, the plasma metabolites and body condition metrics of Blue-footed boobies did not predict breeding continuity in our study. It remains open what explained these contradicting findings, mainly because the studies by González-Medina et al. (2018, 2020) were performed in parallel and, therefore, under similar local food availability and weather conditions, and no predation events were observed at the colony during the study period. The differences are possibly given by unaccounted variation within and among individuals or bias in the selection of individuals in each study (Mitchell et al. 2021). Abandonment of breeding before egg-laying, even when birds were physically prepared to breed, possibly occurred because the individuals were prospecting for potential mates without success (Nelson 1978; Torres and Velando 2010) or were inexperienced or senescent breeders (Kim et al. 2011). In addition, there were no differences in the plasma metabolites between individuals that abandoned breeding after incubation and chick-rearing with those who continued breeding. This result probably occurred as individuals did not reach a physiological limit (which could be measurable by plasma metabolites) and prioritized their body condition before breeding success by abandoning their eggs or chicks. Accordingly, long-lived birds can be expected to operate in normal circumstances and not under the maximal motivation linked to the cost of reproduction (Fowler et al. 2018).

It is worth noting that the current study was performed under favorable breeding conditions related to high prey availability. Between July 2010 and February 2012, there was an extreme La Niña event with many small pelagic fish schools (Rubio-Rodríguez et al. 2018). Also, breeding success was overall good, i.e. the number of failed breeders was much lower than that of successful breeders. However, given that plasma metabolite levels and body mass variation might vary according to foraging conditions (Bauch et al. 2010; Graña Grilli et al. 2018), one can expect the following: (1) our results on plasma metabolites and body condition metrics contrast with years with less favorable conditions that cause birds to be energetically more constrained; (2) that plasma metabolites and body conditions metrics show minor variation between individuals during those years because only individuals with better foraging abilities would breed; or (3) that in particularly poor years, inter-individual variation in plasma metabolites and body condition metrics will be undetectable because individuals abandon breeding before reaching a negative threshold. For example, at Isla Isabel, Mexico, during an El Niño event, only ~ 20% of the total Blue-footed boobies’ colony attempted to breed, and from the pairs that attempted breeding, all chicks died (Wingfield et al. 1999). This result suggests that some Blue-footed boobies might attempt breeding during unfavorable conditions, but fail as they get energetically constrained. However, if conditions are too poor, Blue-footed boobies might not breed at all, as occurred for Blue-footed boobies’ colonies at the Galapagos Island, also during an El Niño event (Wingfield et al. 2018). Based on the present study results, individuals might abandon breeding even before reaching a physiological limit, which could be measurable by plasma metabolites. Therefore, it would be difficult to prove the relationship between plasma metabolites and body condition with environmental variability.

## Conclusion

To our knowledge, this is the first study to report the nutritional state variations of individual tropical seabirds from pre-laying to the late chick-rearing period. We found that only body mass but none of the plasma metabolites were repeatable. Also, individuals exhibit body mass changes (Δ_BM_) according to their sex and breeding stage, but the patterns of Δ_BM_ do not mirror the nutritional state of the breeding individuals. Instead, females showed a decline in body mass at the end of the season, whereas males slightly recovered. The breeding biology of Blue-footed boobies likely explains the pattern found in the nutritional state (using plasma metabolites) between sexes and breeding stages. In this species, females, the larger sex, take over the demands of breeding (egg production and chick-provisioning), whereas males, the smaller sex, are limited in their investment in breeding and might need to avoid carry-over effects for their next breeding season. The breeding demands and each sex roles might also help explain differences in the foraging behavior found in sulids, even in monomorphic species. These results contribute to a better understanding of foraging and breeding decisions in long-lived seabirds.

## Supplementary Information

Below is the link to the electronic supplementary material.

Supplementary file1 (DOCX 111 KB)

## Data Availability

Datasets deposited at Zenodo 10.5281/zenodo.7053242.
