# Supplementary material for: Nutritional state variations in a tropical seabird throughout its breeding season
Source: J Comp Physiol B. 2022 Sep 13;192(6):775–87. doi: 10.1007/s00360-022-01456-3 (PMC9550769; doi:10.1007/s00360-022-01456-3)

# Supplementary Information

**Title:** Nutritional state variations in a tropical seabird throughout its breeding season

**Journal:** Journal of Comparative Physiology B

**Authors:** Miriam Lerma^1,2,^*, Nina Dehnhard, José Alfredo Castillo-Guerrero, Guillermo Fernández

^1^Posgrado de Ciencias del Mar y Limnología, Universidad Nacional Autónoma de México, Ciudad Universitaria, Coyoacán 04510, Ciudad de México, México

^2^Research and Technology Center (FTZ), University of Kiel, Hafentörn 1, 25761 Büsum, Germany

*corresponding author: lerma@ftz-west.uni-kiel.de

**Fig S1** Relationship between ulna size and body mass of individual Blue-footed boobies (*Sula nebouxii*). Females in circles, males in triangles


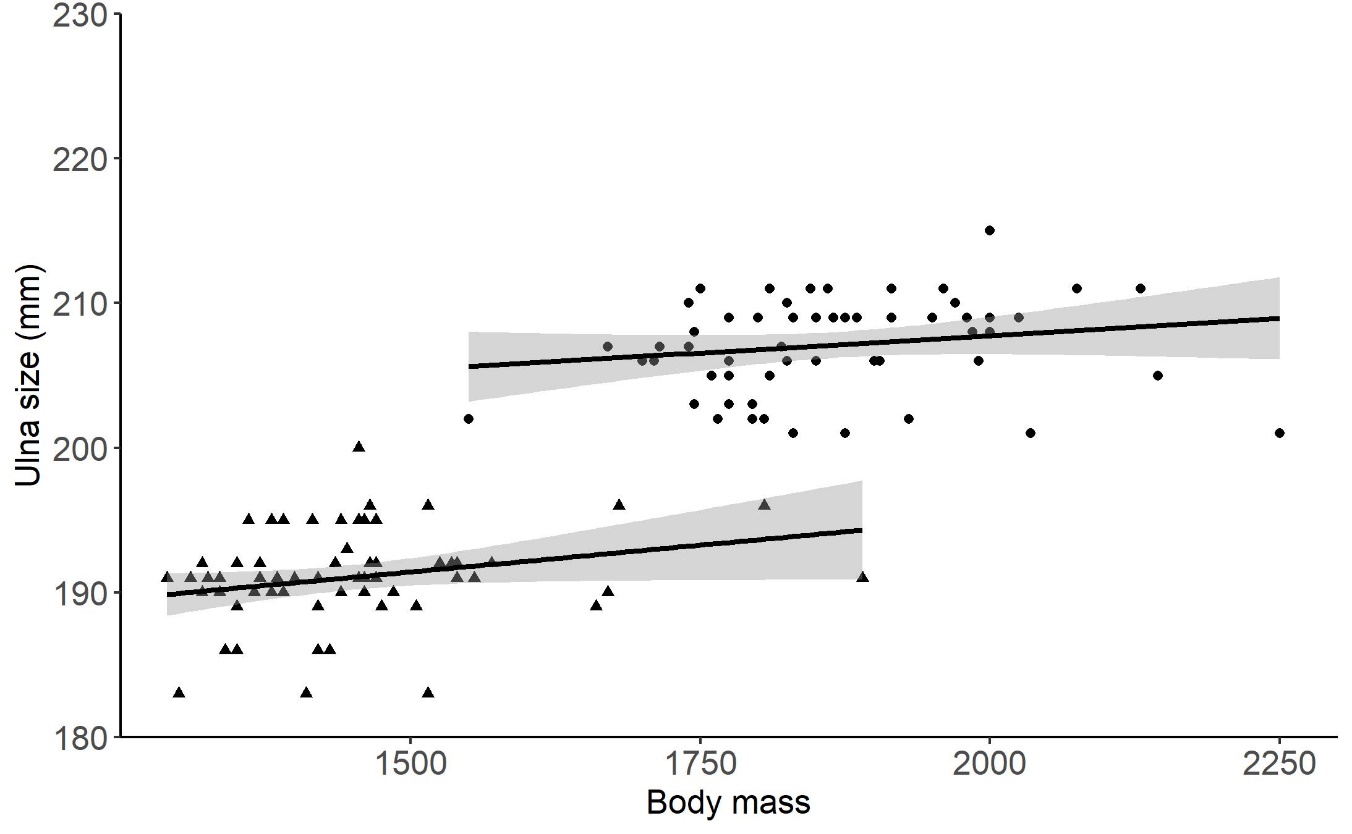

Supplement: Supplementary file 1 — Supplementary file1 (DOCX 111 KB) [file 360_2022_1456_MOESM1_ESM.docx]
